# Feline upper respiratory tract disease – Computed tomography and laboratory diagnostic

**DOI:** 10.14202/vetworld.2022.1880-1886

**Published:** 2022-07-30

**Authors:** Armands Vekšins

**Affiliations:** Clinical Institute, Faculty of Veterinary Medicine, Latvia University of Life Sciences and Technologies, Jelgava, Latvia

**Keywords:** computed tomography, feline, nasal neoplasia, *Mycoplasma felis*, rhinitis

## Abstract

**Background and Aim:**

Upper respiratory tract disease (URTD) is prevalent in cats, and diagnosis can be challenging. This study aimed to determine the most common causes of cat URTD in Latvia and describe computed tomography (CT) and laboratory diagnostic findings.

**Materials and Methods:**

The present retrospective study included a total of 94 cats who were diagnosed with URTD. All cats underwent CT, and 50 of them had additional diagnostic tests, such as histology and respiratory infection polymerase chain reaction (PCR) testing.

**Results:**

The most common CT finding was rhinosinusitis (55.32%) followed by nasal neoplasia (26.6%) and nasopharyngeal polyp (14.89%), but in three cats, a cause of respiratory symptoms was larynx neoplasia, nasal dermoid cyst, and an oronasal fistula. PCR test showed that the most cause of rhinosinusitis was *Mycoplasma felis*. Nasopharyngeal polyp as the primary diagnosis was identified in 14 cats from 3 months to 6 years, with an average age of 1.85 ± 1.915 years, and 54% of cats were female. Nasal neoplasia as a primary CT diagnosis was determined in 25 cats at the age of 5–18 years, with an average age of 10.56 ± 3.416 years. Histology diagnosis included four types of neoplasia – squamous cell carcinoma, sarcoma, adenocarcinoma, and aplastic carcinoma.

**Conclusion:**

This study describes the most common CT and laboratory findings in cats with URTD. Included information will be helpful for general veterinary practitioners and researchers and will update their knowledge on feline URTD.

## Introduction

The most common symptoms of feline upper respiratory tract disease (URTD) are nasal discharge, sneezing, dyspnea, and stridor. The nasal cavity is often affected by respiratory infections, nasal mycosis, nasal parasites, nasopharyngeal polyps (NP), and nasal tumors; however, in some cases, disorders can be bonded to an allergic or idiopathic reaction [1].

Respiratory infections, especially in young cats, play a significant role in feline URTD spread, and cats can be infected by a variety of pathogens, for instance, feline herpesvirus (FHV), *Feline calicivirus* (FCV) [2], and *Bordetella bronchiseptica* [3].

It is undeniable that viruses and bacterial infections have a crucial role in URTD, yet *Mycoplasma* infection is a common cause of upper respiratory symptoms. However, diagnosing *Mycoplasma* spp. is challenging, and a gold standard is the polymerase chain reaction (PCR) test [4]. The previous studies [5, 6] demonstrate that URTD often is caused by coinfections, and prevalent infection agents are *Mycoplasma felis*, FCV, FHV-1, and *Chlamydia*. Even though respiratory infections can affect cats individually, they often outbreak in animal shelters and multi-cat households [7]. In cats with upper respiratory tract infections, secondary can develop NP. Polyps are non-neoplastic, inflammatory growths that develop in the middle ear and extend into the pharynx [8].

Fungal respiratory infections in cats are an orphan disease; however, fungal rhinitis can be caused by aspergillosis, cryptococcosis, hyalohyphomycosis, and trichosporonosis [8]. Aspergillosis occurs worldwide, and it is described in Australia, North America, the United Kingdom, Switzerland, Germany, and other countries. Aspergillosis has sinonasal or sino-orbital form, and clinically cats have sneezing, unilateral or bilateral serous to mucopurulent nasal discharge, and rarely epistaxis [9].

Nasal tumors, especially in elderly cats, are a common URTD cause. Most of them are malignant, with a prevalence of lymphoma and adenocarcinoma. Benign nasal tumors include adenomas, fibromas, papilloma, and transmissible venereal tumors [1, 10].

URTD diagnostic involves laboratory testing and diagnostic imaging. For infectious diseases, real-time PCR analysis is recommended to take a combination of an oropharyngeal swab and either conjunctival or a nasal swab for PCR testing [6]. Fungal infections can diagnose by cytology, histology, or fungal culture. Several Aspergillosis diagnostic tests are described and one of them is detection of Aspergillus antibodies; however, up to 15% of healthy cats are seen as having positive *Aspergillus* antibodies. Therefore, a positive *Aspergillus* antibodies test must be evaluated together with clinical signs [9]. Although in humans’ serum, galactomannan (GM) is measured for early diagnosis of aspergillosis invasion, in cats’ serum, GM measurement has a poor sensitivity [11].

Although many diagnostic imaging methods are available, not all of them are suitable for URTD diagnosis. Nasal neoplasia and rhinitis differentiation can be challenging. Radiographs have poor sensitivity, and rhinoscopy provides limited information. Rhinoscopy can help view the internal structures, identify abnormalities and foreign bodies, and biopsy or take samples for bacterial and fungal cultures [12]. Nasopharyngeal polyp diagnostic includes inspection above the soft palate, rhinoscopy, radiography, CT, and magnetic resonance imaging [13].

CT can aid in differentiating nasal neoplasia versus rhinitis. CT findings of nasal neoplasia include osteolysis of the paranasal bones, turbine destruction, nasal septum lysis, and homogenous mass [14]. Animals with moderate rhinitis can be diagnosed with scattered soft-tissue density, clearly defined turbine margins, and air passages; however, in cases of severe rhinitis, moderate turbine destruction can be seen [15]. Fungal rhinitis is characterized by specific computed tomography (CT) findings, including turbinate lysis, frontal sinus mucosal thickening, soft-tissue attenuating material bilaterally or unilateral in the nasal cavity or frontal sinuses, and paranasal bone lysis [16].

The nasal tumor staging system is adapted from human medicine, and tumors are divided into four stages: Stage 1 tumor is confined in one nasal passage, paranasal sinus, or frontal sinus; Stage 2 includes bony involvement but with no evidence of orbit, subcutaneous or submucosal mass, Stage 3 characterized with orbit, nasopharynx, or subcutaneous or submucosal mass, but Stage 4 tumor with extension into the cribriform plate. Cassie N. Lux and others describe an advanced imaging tumor staging system, where the stage is granted regarding the previously described Word Health Organization nasal tumor staging system parameter number; for instance, Stage 2 includes one of the parameters listed before, but Stage 4 is characterized by the presence of all criteria [17].

Therefore, this study aimed to determine the most common causes of cat URTD in Latvia and describe CT findings.

## Material and Methods

### Ethical approval

Ethical approval was not required to conduct this study.

This study was carried out from January 2020 to December 2021 at Latvia University of Life Sciences and Technologies (LLU) Veterinary Hospital.

### Study design

The present retrospective study included a total of 94 cats who were diagnosed with URTD. All cats’ underwent CT and 50 of them had additional diagnostic tests, and those are, histology, PCR tests, bacteriology, and cytology.

### Computed tomography

Computed tomography was performed in the LLU Veterinary Hospital with a 16-slice multidetector Philips MX-16 CT scanner (Philips, Netherland). During the examination, cats were positioned in sternal recumbency. High-resolution CT scans of the head region were performed. Native and post-contrast (Ultravist 623 mg/mL [300 mg/mL iodine], 2 mL/kg IV) 2 min delayed phase were obtained. Standard bone (WL 400; WW 3000) and soft-tissue (WL 50; WW 300) window algorithms were used. Cats were eligible for this study if CT confirmed the diagnosis of inflammatory changes (rhinitis, sinusitis, and NP) and nasal or sinonasal neoplasm. For cats with complex findings, neoplasia was considered as the main cause of URTD.

### Histology

Samples for histology were obtained with biopsy forceps through nostrils or were taken during the surgery. Histological examination was done by the Diplomate of the American College of Veterinary pathologists.

###  Polymerase chain reaction

Polymerase chain reaction tests for FHV, *M. felis*, *B. bronchiseptica*, and FCV were performed if CT findings confirmed an inflammation.

### Statistical analysis

Statistical analysis was performed using MS Excel software version 2013 (Microsoft Office, USA). Descriptive statistics and correlation analysis were used. A statistically significant correlation was considered if the probability value (p) was less than 0.05.

## Results

**Figure-1:**
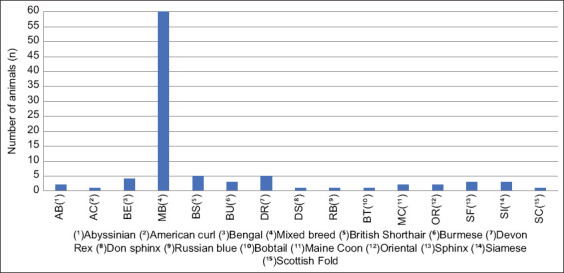
Included cat breeds (%).

Animal age was from 3 months to 18 years, with an average age of 6.15 ± 4.716 years; 59 were male, but 35 female cats, and seventy were neutered. Different breed cats were included (Figure-1); however, the mixed breed was the majority.

Felines were classified according to the primary CT diagnosis (nasal neoplasia, rhinosinusitis, nasopharyngeal polyp, or another diagnosis). The principal CT diagnosis was rhinosinusitis (n = 52) followed by nasal neoplasia (n = 25) and nasopharyngeal polyp (n = 14); however, three cats cause of respiratory symptoms was larynx neoplasia, nasal dermoid cyst, and an oronasal fistula.

### Rhinosinusitis

**Figure-2:**
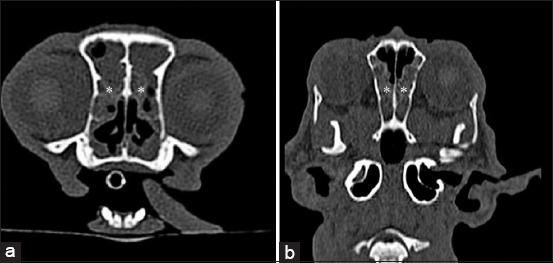
(a) Transverse and (b) coronal computed tomography images of rhinosinusitis. Large amount of soft-tissue/fluid density (asterisk).

As the primary CT finding, rhinosinusitis (Figure-2) was diagnosed in 52 cats from 5 months to 14 years, with an average age of 4.63 ± 3.818 years, and 56% of cats were male and in 43 cases, additional diagnostic tests were used - PCR test (n = 27), histology (n = 10), other tests (n = 6).

PCR analysis with 66.6% positive test showed that the most common infection was *M. felis*. PCR test results are presented in Table-1. Two of the tested animals had multi-infection. The first case of a 4-year-old mixed-breed male cat with a CT diagnosis of unspecific bilateral rhinitis was positive for *M. felis* and herpesvirus and the second case was a 10-month-old Bengaline cat who was positive for calicivirus, herpesvirus, *B. bronchiseptica*, and *M. felis*.

**Table 1:** Respiratory infection PCR test results.

PCR test	Total number of animals with rhinosinusitis (n)	Number of tested animals (n)	Positive result against total number (%)	Positive result against tested animals (%)
*Mycoplasma felis*	52	27	20	40.74
Herpesvirus			10.91	22.22
Calicivirus			1.81	3.7
*Bordetella bronchiseptica*			1.81	3.7

PCR=Polymerase chain reaction, *B. bronchiseptica=Bordetella bronchiseptica*

The most common histological diagnosis was lymphoplasmacytic rhinitis (n=5; 50% of tested animals) and in 80% of cases, it was chronic. Histology results are presented in Table-2.

**Table 2:** Histology results of cats with CT confirmed rhinitis and rhinosinusitis diagnosis.

Feline breed, gender, and age	CT diagnosis	Histology results
Mixed breed, female, 8 years	Nasal abscess (differential diagnosis-neoplasia), bilateral rhinosinusitis, bilateral otitis media, right medial retropharyngeal lymphadenopathy.	Rhinitis (suppurative, lymphoplasmacytic, chronic) with bacteria and yeast organisms (*Candida* spp.).
Mixed breed, male, 4 years	Destructive rhinitis (differential diagnosis: Neoplasia).	Lymphoma (large cell, diffuse, low grade), rhinitis (suppurative, lymphoplasmacytic).
Maine coon, male, 12 months	Rhinosinusitis	Rhinitis (suppurative, eosinophil, lymphoplasmacytic, subacute, severe, with necrosis).
Mixed breed, male, 9 years	Rhinitis	Rhinitis (mucopurulent, chronic, severe with epithelial ulceration)
Burmese, male, 5 years	Rhinosinusitis, lymphadenopathy	Rhinitis (suppurative, lymphoplasmacytic, chronic) with bacteria (*Actinomyces* or *Nocardia* spp.).
Abyssinian, female, 6 months	Rhinosinusitis	Rhinitis (suppurative, lymphoplasmacytic, chronic).
Mixed breed, female, 9 years	Destructive rhinosinusitis (differential diagnosis: Neoplasia)	Lymphoma (medium to large cell, diffuse, low grade) rhinitis (lymphocytic and granulomatous, multifocal, chronic, epithelial attenuation).
British shorthair, female, 8 years	Unilateral destructive rhinosinusitis (differential diagnosis: Neoplasia, fungal infection)	Rhinitis (suppurative, chronic, severe, fungal infection – *Aspergillus* spp.).
Devon Rex, male, 9 months	Unilateral destructive rhinitis (differential diagnosis: Fungal infection, nasal hematoma)	Rhinitis (suppurative and plasmacytic, chronic, severe with mucopurulent and fibrinous exudate).
Mixed breed, male, 12 years	Non-specific rhinitis, bilateral otitis media.	Rhinitis (lymphoplasmacytic, chronic, moderate). Without bacterial and fungal infection.

CT=Computed tomography

In addition to the main diagnosis, two cats also had middle ear inflammation.

### Nasopharyngeal polyp

**Figure-3:**
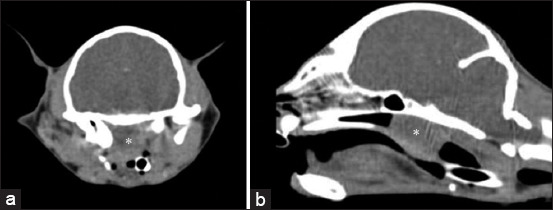
(a) Transverse and (b) sagittal computed tomography images of nasopharyngeal polyp. Nasopharyngeal meatus is obligated by soft-tissue density (asterisk).

Nasopharyngeal polyp (Figure-3) as the primary CT diagnosis was diagnosed in 14 cats from 3 months to 6 years, with an average age of 1.85 ± 1.915 years, and 54% of cats were female and in three cases diagnosis was confirmed histologically. Nasopharyngeal polyp prevalence rate in a mixed-breed population was 75%.

### Nasal neoplasia

**Figure-4:**
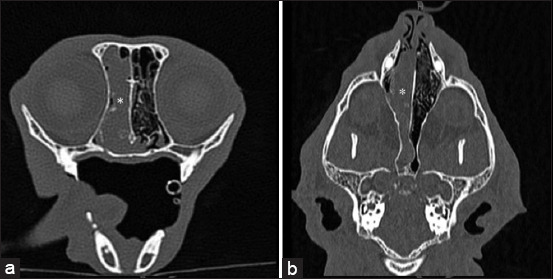
Transverse (a) and coronal (b) computed tomography images of nasal neoplasia. Nasal cavity is filled with soft-tissue density (asterisk).

Nasal neoplasia as a primary CT finding was diagnosed in 25 cats aged 5–18 years, with an average age of 10.56 ± 3.416 years. Twenty (80%) were male and 5 (20%) female cats. Clarifying the diagnosis of CT suspected nasal neoplasia, a histological examination (n=6) and cytology (n=1) were performed. Four nasal tumors were confirmed histologically, i.e., squamous cell carcinoma, sarcoma, adenocarcinoma, and aplastic carcinoma. CT findings of nasal neoplasia is presented in Figure-4.

### Other findings

**Figure-5:**
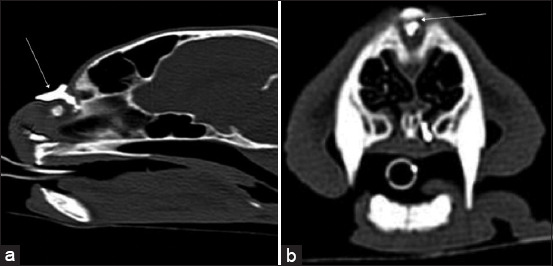
Sagittal (a) and transverse (b) computed tomography images of dermoid cyst. A fistulogram shows a dorsal opening of the nasal planum (arrow).

**Figure-6:**
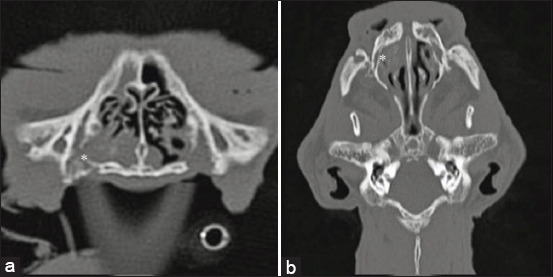
Transverse (a) and coronal (b) computed tomography images of oronasal fistula and fluid density in a nasal cavity (asterisk).

**Figure-7:**
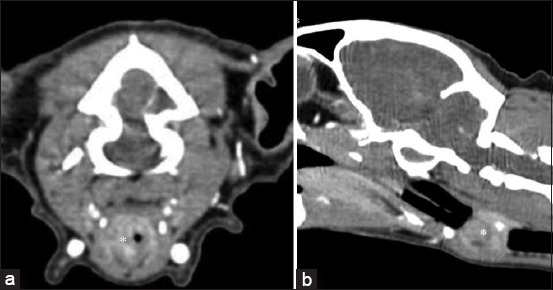
Transverse (a) and sagittal (b) computed tomography images of laryngeal neoplasia (asterisk).

Three cats with URTD were diagnosed with a dermoid cyst, oronasal fistula, and larynx neoplasia. A 2-year-old mixed-breed female cat with nasal discharge from kitten age had a dermoid cyst (Figure-5). Rhinosinusitis secondary to oronasal fistula (Figure-6) was diagnosed in a 15-year-old British shorthair male cat. In a 13-year-old Devon Rex female cat, respiratory symptoms were not due to nasal cavity disease, but CT confirmed laryngeal neoplasia (Figure-7). The correlation between CT diagnosis and animal age was r = 0.51, p < 0.01, and animal gender r = 0.17, p > 0.05.

## Discussion

The study results showed that URTD affects cats of different ages and breed. Therefore, for each patient, a diagnostic plan must be based on the cat’s age, history, symptoms, and the results of previously performed diagnostic and treatment.

Computed tomography is an excellent diagnostic imaging method for skull examination; however, it has several limitations, and for final diagnosis additional tests might be necessary. This study presents the most common CT and laboratory diagnostic findings in cats with URTD.

This study included animal age range from 3 months to 18 years. Results showed that NP more commonly affects young animals (1.85 ± 1.915 years), rhinosinusitis affects medium age (4.63 ± 3.818 years), and nasal neoplasia affects elder cats (10.56 ± 3.416 years). Correlation analysis showed a significant relationship between animal age and CT findings. This study results showed that male cats more commonly suffered from URTD and that coincide with other author studies, for example, Dinnage and others found out that neutered and castrated male cats had higher URTD rates than females [18]. However, correlation analysis did not show a significant link between gender and CT diagnosis.

A study conducted by Dinnage *et al*. [18] shows ambiguous results of URTD commonly affected cat breeds. Dinnage and others conclude that purebred cats have URTD higher risk than mixed breed; however, our study presents opposite results because 63.83% of cats were mixed breed. Another view that coincides with this study’s results is expressed by Sykes *et. al*. [19] and consider that mixed-breed cats are more likely to be positive for respiratory infections, such as FHV1. Since Latvia is not collected data about cats, it bothers to evaluate and compare URTD affected cat correlation with the total population.

Based on CT findings, rhinosinusitis was a principal diagnosis. Diagnosis was set for cats in quite wide age (5 months–14 years) and that was the reason why addition diagnostic procedures differed. PCR tests were chosen for younger cats, and results showed that the most diagnosed infection was *M. felis*. Results coincide with the previous studies, pointing out that *M. felis* is a significant cause of URTD [5]. Two of the tested cats had a multi-infection. A study conducted by Cohn [20] shows that young cats with respiratory infections common have multiple infections simultaneously, and regular tandem is *M. felis* and FCV. It should be noted that in rare cases, rhinitis can be caused by atypical pathogens, for instance, *Leishmania* spp. [21], therefore URTD diagnostic plan must be prepared individually, regarding each patient’s individual parameters and current diseases of a certain region.

Chronic lymphoplasmacytic rhinitis was the most diagnosed type of rhinosinusitis. Lymphoplasmacytic rhinitis is a rare disease characterized by lymphoplasmacytic inflammation and progressive tissue destruction. The previous study conducted by Roccabianca *et al*. [22] showed that cats with lymphoplasmacytic rhinitis were at the age of 11 years; however, that does not match our results because lymphoplasmacytic rhinitis was diagnosed in cats from 6 months to 12 years with a median age of 5.3 years. Although viral and bacterial infections are the most common causes of URTD, for one cat, *Aspergillosis* spp. infection was diagnosed. The patient was an 8-year-old British shorthair cat, and CT findings included unilateral destructive rhinosinusitis. Nasal aspergillosis more often affects dogs, and in cats’, fungal infections are reported less frequently [23]. Nasal aspergillosis treatment is complicated and disseminated diseases may develop [24]. In cases when CT findings include destructive rhinitis or rhinosinusitis, it is important to include nasal tumors or fungal infection as differential diagnosis. In our study, two cats with differential diagnosis of destructive rhinosinusitis included nasal neoplasia and that was histologically confirmed, and lymphoma was detected.

Nasopharyngeal polyps are inflammatory masses that arise from the mucosa of the nasopharynx of the tympanic membrane. Even though NP are more commonly diagnosed in young cats [25], studies show that cat age range with this disease can be from 3 months to 16 years [26]. In our study, 14 cats had NP with a median age of 1.85 years, and most of them were mixed breed.

Nasal neoplasia was diagnosed in 25 cats and in six cases diagnosis was confirmed histologically. Four types of nasal neoplasia were diagnosed, i.e. squamous cell carcinoma, sarcoma, adenocarcinoma, and aplastic carcinoma. A study conducted by Mukaratirwa *et al*. [27] reported that in 92% of cases, nasal neoplasia is malignant, and adenocarcinomas and squamous cell carcinomas are the most commonly diagnosed tumors that coincides with this study results. Malignant nasal tumors typically grow slowly, and most do not have detectable metastasis at the time of initial diagnosis. Nasal neoplasia and rhinitis can be diagnosed by combining CT features and evaluating medial retropharyngeal lymph nodes changes [28]; however, in our study, both cats with CT diagnosis of rhinosinusitis, but histologically detected nasal lymphoma, CT results showed medial retropharyngeal lymph nodes within normal limits. That can be explained by the fact that metastasis in the regional lymph nodes usually is late and even less often in the lungs.

Although respiratory infections, nasopharyngeal polyps, and nasal neoplasia were the most significant and frequent causes of URTD symptoms, our study shows that other causes, such as a dermoid cyst, oronasal fistula, and larynx neoplasia should be included as a possible cause of this disease. Currently information about nasal dermoid cysts is limited, but it is known that cysts evolve because of abnormal embryogenesis during the development of the frontonasal region, and may be demarcated with intracranial extension [29].

## Conclusion

To the author’s knowledge, this is the first study that describes URTD distribution in cats in Latvia, and the study divulges most common CT and laboratory diagnostic findings in cats with URTD. The outlined results will be helpful for all veterinary practitioners and will update their knowledge on feline URTD. Although the study has several limitations, for example, not all patients had the same diagnostic process, the paper still gives valuable and appropriate information. A deeper understanding of URTD causes could be obtained by increasing the number of laboratory and histology testing.

## Author’s Contributions

AV: Conceptualization, methodology, and drafted and revised the manuscript.
